# Concurrent datasets on land cover and river monitoring in Fukushima decontaminated catchment during 2013–2018

**DOI:** 10.1038/s41597-023-02452-0

**Published:** 2023-08-21

**Authors:** Bin Feng, Yuichi Onda, Yoshifumi Wakiyama, Keisuke Taniguchi, Asahi Hashimoto, Yupan Zhang

**Affiliations:** 1 Center for Research in Radiation, Isotopes, and Earth System Sciences, University of Tsukuba, Tsukuba, 305-8572 Japan; 2 Institute of Environmental Radioactivity, Fukushima University, Fukushima, 960-1296 Japan; 3 National Institute of Technology, Tsuyama College, Tsuyama, 708-8509 Japan

**Keywords:** Environmental impact, Geomorphology, Hydrology, Element cycles, Sedimentology

## Abstract

After the Fukushima nuclear accident, the Japanese government implemented extensive decontamination work in ^137^Cs contaminated catchments for residents’ health and local revitalization. Whether dramatic land use changes in the upstream decontaminated regions affected river suspended sediment (SS) and particulate ^137^Cs discharge downstream remain unknown because of the poor quantification on land cover changes and long-term river SS dynamics. We here introduce a 6-year concurrent database of the Niida River Basin, a decontaminated catchment, including the first available vector decontamination maps, satellite images in decontaminated regions with a spatial resolution of 10 m, and long-term river monitoring datasets spanning decontamination (2013–2016) and subsequent natural restoration stages (2017–2018). These datasets allow us, for the first time, to directly link the transport dynamics of river SS (particulate ^137^Cs) to land use changes caused by humans in real-time, which provide fundamental data for better understanding the river response of sediment to land use change. Moreover, the data obtained by interdisciplinary methods offer a template for land use change impact assessment in other river basins.

## Background & Summary

Fukushima nuclear accident that occurred on 11th March 2011 in Japan is one of the worst nuclear disasters in human history recognized by the International Atomic Energy Agency (IAEA)^1^, which resulted in a release of over 520 PBq (1PBq = 10^15^ Bq, excluding noble gases) of radionuclides from the damaged Fukushima Daiichi Nuclear Power Plant (FDNPP) into the environment^2^. Among the numerous FDNPP-derived radionuclides, Cesium-137 (^137^Cs) triggers a specific concern owing to its relatively long half-life (T_1/2_ = 30.11 years) and high radiation risk (gamma emitter)^3–6^. Recent estimates suggest that approximately 12–62 PBq of radiocerium were leaked during the nuclear accident^7^, in which approximately 2.7 PBq of ^137^Cs were dispersed in the terrestrial environments via the dry and wet deposition^8^. The air dose rates at 1 m above ground in some contaminated regions were immediately raised to a level two orders of magnitude higher than the background in Japan due to the high ^137^Cs contamination^9,10^. Moreover, terrestrial ^137^Cs contamination is slowly expanding to distal regions as it can get tightly bound to the surface eroded soil and transported distally (e.g., the Pacific Ocean) through rivers^11^.

To reduce the radiation risk and revitalize the agricultural activities in those contaminated regions, the Japanese government evacuated the residents after the accident and promulgated the preliminary decontamination guideline at the end of 2011 for the intensive contamination area where the annual exposure dose exceeded one mSv^12^. During the decontamination work, contaminations on impermeable surfaces in residential regions were removed by manual methods, including wiping, brushing, high-pressure water washing, and scraping^2^. Contrastingly, mechanical decontamination (or called physical replacement decontamination) was used in the agricultural land and grassland where the surface contaminated soil (about 5 cm), as well as the vegetation, were removed and then replaced with uncontaminated soil^2^. In this case, the vegetation cover in these non-residential regions was thought to be significantly altered during land decontamination. After the decontamination, the governmental project report confirmed the productive results of decontamination in significantly reducing the air dose rate in the decontaminated regions^7^.

When the major attention of the Japanese government and public was focused on the effectiveness of decontamination in reducing regional radiation exposure risk, little attention was paid to whether the dramatic land cover changes in the decontaminated regions would affect the erosion pattern in the watershed and the resulting changes in the downstream discharge of suspended sediment (SS) and particulate ^137^Cs. Given that the sediment in the terrestrial environment represents a critical carrier for nutrients (e.g., carbon^13^, nitrogen^14^, and phosphorus^15^) and environmental pollutants (e.g., radioactive contaminants^16^, heavy metals^17^, and plastics^18^), it may have substantial effects on the riverine trophic structure^19^, elemental cycles^20^, and biodiversity^21^ when a large amount of sediment is transported into the river system. Studying the dynamic relationship between the land cover changes caused by anthropogenic perturbations and fluctuations in river SS load has been a topic of wide interest. Over the past decades, although numerous river monitoring campaigns have been conducted in different scale catchments around the world^22–24^, it is still difficult to evaluate the influence of a specific perturbation because multiple perturbations may simultaneously exist in the catchment, and their duration varies significantly. Even though decontamination is almost the only strong anthropogenic perturbation in the Fukushima contaminated regions due to the residents’ evacuation^2^, there remain great challenges in accurately quantifying the land cover changes in the decontaminated region and linking them with concurrent dynamics in suspended sediment load in the river system owing to the uncertainties in the boundary of the perturbation and the lack of long-term and high-resolution river monitoring datasets^12,25–27^.

In this study, we introduce a comprehensive database on the Niida River Basin, a typical decontaminated watershed (area: approximately 265 km^2^) in Fukushima. The details of this database are being reported for the first time, which includes vector decontamination maps, quantified land cover changes in the decontaminated regions based on high-resolution (10 m × 10 m) satellite maps (667 images), and the concurrent river monitoring datasets (water discharge: 440940 records; suspended sediment concentration: 440940 records; particulate ^137^Cs concentration: 68 records) in upstream and downstream catchments spanning the decontamination stage (2013–2016) and subsequent natural restoration stage (2017–2018). Satellite images and river monitoring datasets are available at the Environmental Radioactivity Research Network Center (ERAN, https://www.ied.tsukuba.ac.jp/ernc/en/welcome-to-eran/)^28–31^. This database allows us, for the first time, to quantitatively assess the long-term impacts of dramatic land cover changes on the patterns of river sediment supply and transport, which would provide fundamental information for a better understanding of the response of river SS to land cover changes in the terrestrial environment. Besides, the impacts of upstream land remediation on the environmental sustainability of downstream catchments revealed by our data may offer a valuable lesson for the contaminated catchments waiting for mechanical remediation in the future. In addition, our database holds promise for reuse in research related to soil erosion model validation, river sediment supply and location dynamics. More importantly, we introduced more details about data generation and validation procedure for this database, which could be used as a research framework to analyse regional sediment transportation during intense land cover changes in other river basins worldwide.

## Methods

### Study regions

**Fig. 1:**
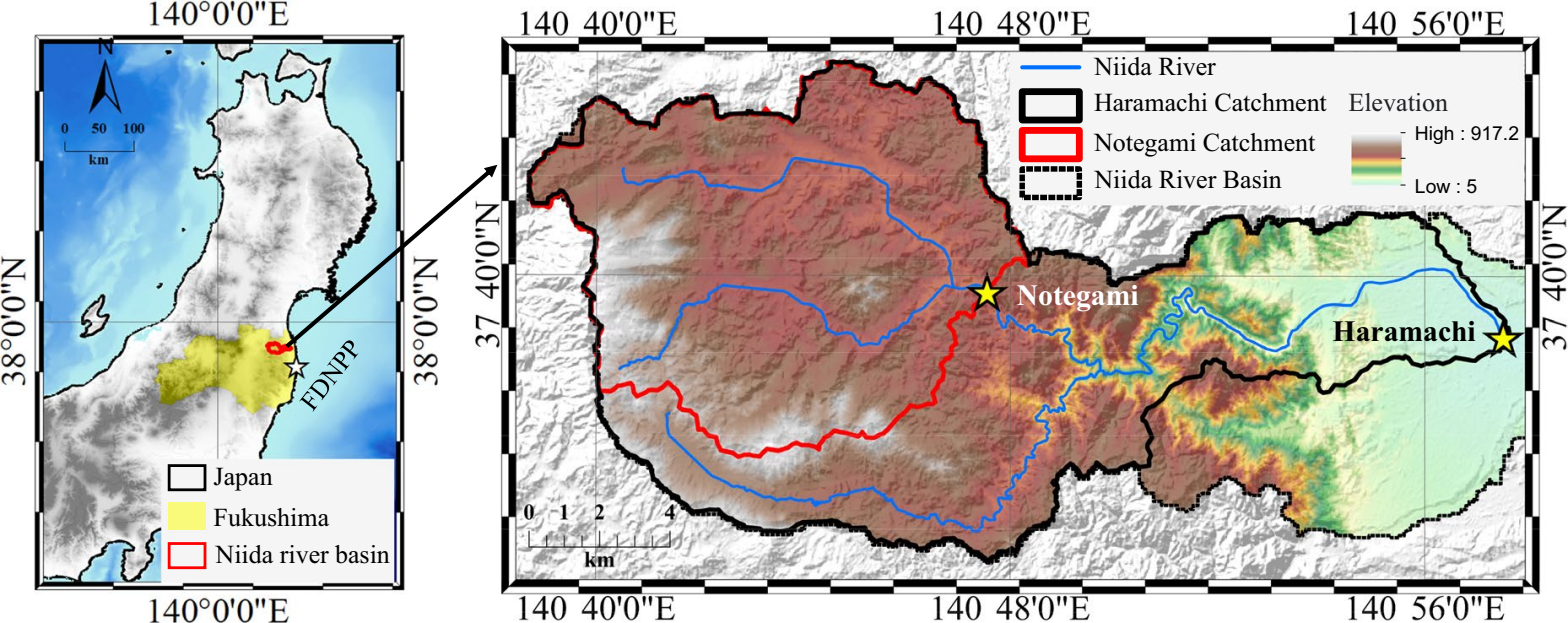
Niida river basin in Fukushima Prefecture, Japan. The two stars with yellow color are the river monitoring station in upstream (Notegami) and downstream (Haramachi), respectively. The elevation information is derived from the DEM map with a spatial resolution of 10 m in the Japanese Geographical Survey Institute.

The Niida River Basin is located in the Fukushima Prefecture, Japan (Fig. 1), approximately 40 km from the damaged FDNPP. The elevation of the study catchment spans a range of 912.2 m, from the minimum of 5 m at the downstream outlet to a maximum of 917.2 m at the upstream boundary. In the upstream region, the mountainous plateau is the dominant topography (altitude: 700–900 m), and the major soil types are the Cambisols and Andosols. The downstream region is a coastal plain in which Fluvisols dominate the soil type. Local meteorological data show that the average annual precipitation in this watershed is approximately 1300 mm, and the main rainy season is typically from May to October each year^32^. Some residential and agricultural regions are distributed in the upstream and downstream catchments, while the ^137^Cs inventory upstream was reported to exceed 1000 kBq m^−2^ ^33^ Such high radiation exposure risk resulting from the served ^137^Cs contamination drove the Japanese government to implement upstream decontamination from 2013 to 2016 (1% decontamination work extended to early 2017)^31^. As a result, regional land cover (vegetation cover) has changed dramatically with a decline from approximately 72% before August 2013 to 59% in August 2016^32^.

### Vector decontamination map

**Fig. 2:**
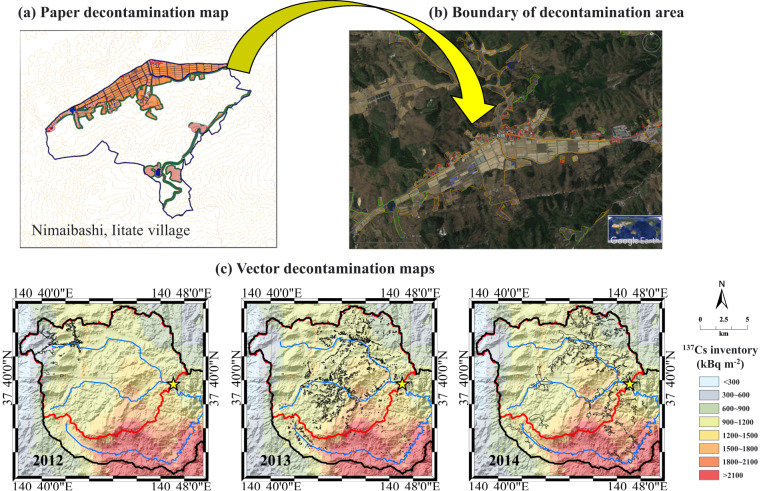
The schematic diagram of the conversion process from paper decontamination maps to vector decontamination maps. (**a**) An example of a decontamination region in Nimaibashi, Iitate village, Fukushima in the original paper map. (**b**) The boundary of this decontamination region in Google Earth Pro. (**c**) The vector decontamination map based on the combination of the decontamination regions in 2012, 2013, and 2014. The ^137^Cs inventory map was derived from Kato *et al*.

To accurately determine the boundary of the contaminated regions, the specific land to be decontaminated was outlined by vector polygons in Google Earth Pro (Fig. 2) based on paper maps of the ordering decontamination area obtained from the Ministry of the Environment, Japan. According to the land use type of the decontaminated region, each polygon was reclassified into the following land use categories: agricultural land, grassland, forest, water body, and residential land. Considering that the ordered decontamination area were updated in 2012, 2013, and 2014, respectively, we integrated all outlined areas annually using Google Earth Pro (KML format) based on the time information of the paper maps (2012: 174 regions; 2013: 1411 regions; 2014: 291 regions) and then converted these KML maps to vector decontamination maps (shapefile format) for each year (Fig. 2) in ArcMap (version 10.3). The ordering decontamination region area is estimated at 2.2, 9.3, and 12.1 km^2^ in 2012, 2013, and 2014, respectively. To better illustrate the decontaminated maps, the World Geodetic System 1984 (WGS 1984) was used as the geographic coordinate system, and the WGS 84/UTM zone 54 N was used as the projected coordinate system.

### Quantification of the land use changes in the decontaminated regions

To quantify the land cover changes within the boundary of decontaminated regions, remotely sensed data were used to calculate the normalized difference vegetation index (NDVI), which is a widely used index to quantify the density of vegetation on the land surface^34^. However, because of technology and budget constraints, acquiring satellite images with high spatial resolution in a short revisit cycle is challenging^35^. For instance, the Moderate Resolution Imaging Spectroradiometer (MODIS) can provide high temporal resolution and cloud-independent satellite products that image is taken for the same area daily, and the highest quality images are displayed over a 16-days period, but its spatial resolution is typically coarse (250 m). Although the satellite images obtained from Sentinel 2 allow us to study the land cover of the terrestrial environment at a high spatial resolution of 10 m, the potential inability to fully cover the study region during the revisiting cycle and the loss of clarity due to the atmospheric contamination or cloud coverage^36^ together result in a much lower temporal resolution of this satellite product than expected. Therefore, improving the resolution of satellite images before NDVI estimation to acquire more information on land-cover changes, is essential.

The enhanced spatial and temporal adaptive reflectance fusion model (ESTARFM) is a recently developed technique that enables the acquisition of images with a high spatiotemporal resolution by pairwise fusion of remotely sensed products from different sensors and has been widely used in monitoring land cover dynamics currently^34^. The key idea of this method is to employ a linear mixture model to blend multi-source data and minimize the system biases to improve fusion performance in heterogeneous areas. By using pairs of fine (i.e., high spatial resolution) and coarse (i.e., low spatial resolution) satellite images acquired in the same period, it is possible to obtain the remote sensing data products with high spatial resolution and frequent coverage from multisource satellite data.

**Fig. 3:**
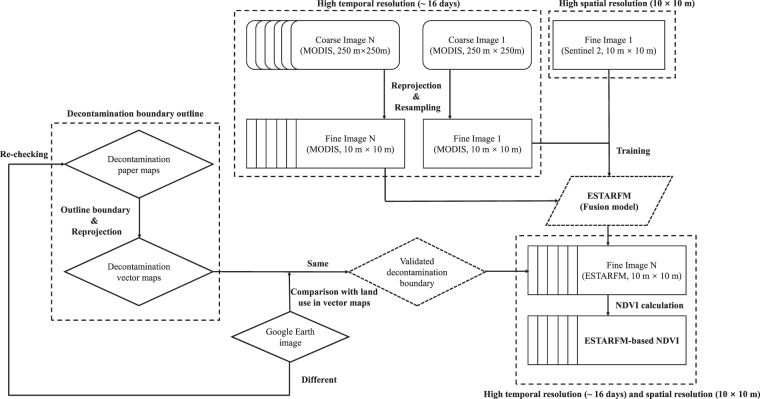
The flowchart of the data-processing procedure in this study. The ESTARFM model was run by the code reported by Zhu *et al*.

In this study, we used the operational flowchart described by Zhu *et al*.^35^ to fuse the satellite maps obtained by MODIS and Sentinel 2 to generate a new set of images with the same temporal resolution as MODIS and the same spatial resolution as Sentinel 2. Fig. 3 illustrates the flowchart of the data-processing procedure. Briefly, the red and near-infrared band images of a MODIS map (i.e., coarse images, spatial resolution: 250 m) downloaded from the National Aeronautics and Space Administration (NASA) reverb (https://search.earthdata.nasa.gov/search) were first merged into a map in ENVI software (version 5.4) for fusion preparation. Resampling were then performed for the prepared MODIS image in ENVI software to set the spatial resolution of MODIS to be the same as that in Sentinel 2 (i.e., fine images, spatial resolution: 10 m). Subsequently, satellite images derived from the United States Geological Survey, (USGS; https://earthexplorer.usgs.gov/) were fused with the time-matched MODIS image in the IDL software (version 5.4). It should be noted that the sizes and geographic locations of the paired satellite images should be cropped to the same before running the code. As the Sentinel 2 images were only available after 2016 in this study, we excluded the cloud-contaminated and incomplete satellite images and used images taken in April and December 2016 for the initial fusion. Finally, based on the available MODIS maps, we generated a new dataset of satellite images with a spatial resolution of 10 m from 2011 to 2018.

**Fig. 4:**
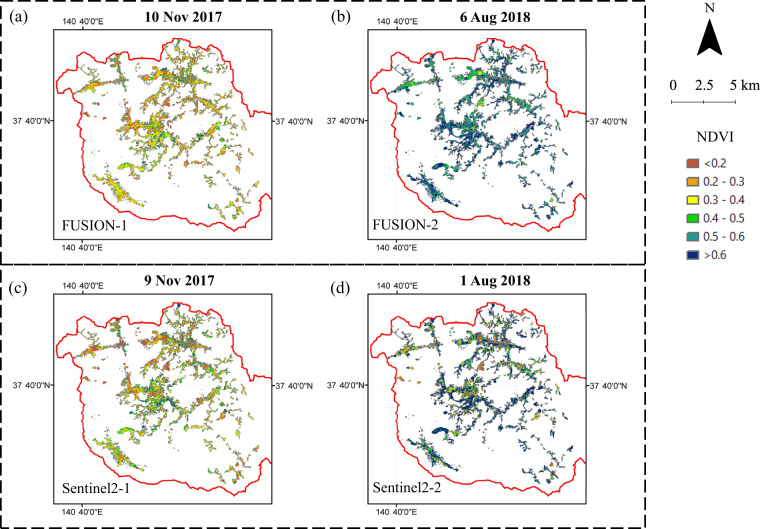
Comparison in NDVI maps calculated using satellite images from the ESTARFM model (i.e., FUSION) and Sentinel 2 during the same period. In the winter season, ESTARFM-based NDVI (**a**, 10 Nov. 2017) and Sentinel 2-based NDVI (**c**, 9 Nov. 2017) were used as an example for comparison. In the summer season, ESTARFM-based NDVI (**b**, 6 Aug. 2018) and Sentinel 2-based NDVI (**d**, 1 Aug. 2018) were taken into comparison.

The NDVI of the newly generated satellite image was estimated using the following equation^37^.

1 $$NDVI=\frac{NIR-R}{NIR+R}$$

where *NIR* and *R* represent the spectral reflectance datasets in the red (*R*, nm) and near-infrared (*NIR*, nm) regions, respectively. By using the boundary of the ordered decontamination maps in 2012, 2013, and 2014, the NDVI images for specific ordered years and whole decontamination regions were further prepared. Subsequently, the data quality assessment was applied to all NDVI images, and pixels were excluded if the NDVI value exceeded the range of −1 to 1. Fig. 4 illustrates an example of the NDVI maps calculated by the satellite images from Sentinel 2 and the ESTARFM model during the same period (winter and summer). Finally, linear interpolation was performed on the mean NDVI value obtained from ESTARFM-based images to generate daily land use change curves, which can be further used to quantitively link the transport dynamics of river SS and particulate ^137^Cs.

### Concurrent river monitoring campaign

To continuously record the fluctuations in water discharge and suspended sediment concentration in the Niida River Basin, a long-term monitoring campaign was conducted in the upstream catchment (Notegami) during 2015–2018 and downstream catchment (Haramachi) during 2013–2018 (Fig. 1, yellow stars). An automatic monitoring station, mainly consisting of a water level sensor (Rugged TROLL100 Data Logger, USA) and a turbidity sensor (ANALITE turbidity NEP9530, McVan Instruments, USA), was set in each monitoring site. Solar panel and rechargeable batteries were prepared to provide sufficient electric energy for instrument operation. Recorded data were collected by our collaborator every month to a quarter. Based on the calibrated water level-discharge equation and the turbidity-SS concentration equation, the water level and turbidity datasets at a temporal resolution of 10 min were converted to the corresponding discharge (*Q*, m^3^ 10 m^−1^) and suspended sediment concentration (*C*, g L^−1^).

**Fig. 5:**
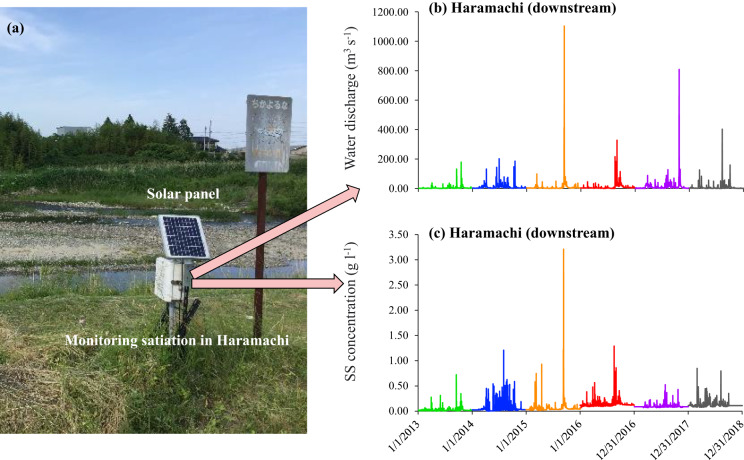
The example of a river monitoring station. (**a**) Field picture of river monitoring station in Haramachi site. (**b,c**) were the temporal variation in water discharge and SS concentration in Haramachi from 2013 to 2018 with a resolution of 10 min.

As monitoring sensors are vulnerable to the environment, frequent flooding and moss interference in monitoring rivers inevitably affect the data recording process, resulting in some missing or abnormal data. Therefore, we reorganized the raw monitoring datasets based on the analysis procedure established in our laboratory previously^27^. Briefly, the water-level data derived from Fukushima prefecture’s official monitoring network were used to fill in the gaps in the water-level data. Regarding the turbidity dataset, we first used the HEC-DSS Vue to automatically check for outliers among the recorded turbidity datasets. Manual examination was then applied to the checked datasets by comparing the discharge and turbidity dynamics. The turbidity data were removed if the value significantly exceeded the highest value of the given year or if the value was considerably higher than the before and after data values. Linear interpolation was used to fill in the data gap for sporadic missing data. When there was a large amount of problematic turbidity data, we estimated the suspended sediment concentration directly from the water discharge at the same time based on the annual rating curve (corresponding to the specific year) between available water discharge and SS concentration (power function). Owing to the lack of an official water-level gauge upstream of the Niida River Basin, we did not perform data filling and estimation for this station. Eventually, we obtained a total of 881880 records of water discharge and SS concentration upstream during 2015–2018 (250872 records) and downstream during 2013–2018 (631008 records). Taking the downstream monitoring site as an example, Fig. 5 shows an actual site picture and the monitoring results.

To obtain the river particulate ^137^Cs dynamics in decontaminated catchments, the passive samplers proposed by Phillips *et al*.^38^ were deployed upstream (Notegami and Warabi) and downstream (Haramachi and Sekekawa) for the time-integrated collection of river SS. Table 1 summarizes the sampling information for each catchment. At each site, the passive sampler was installed at a certain depth of 20–30 cm above the riverbed. After a few months of deployment (typically 1–3 months), our field collaborator transferred the collected SS samples and turbid water from the sampler into a clean water container. In the laboratory of the Institute of Environmental Radioactivity, Fukushima University, we separated the river water and SS samples and dried the collected SS samples in an oven at 105 °C for 24 h. The ^137^Cs activity in the dried SS sample was determined by high-purity germanium gamma-ray spectrometry (GCW2022S, Canberra−Eurisys, Meriden, USA) that was calibrated using standard soil samples originating from IAEA. Counting often takes approximately 1–24 hours until an acceptable measurement uncertainty of less than 10% is achieved. Decay correction was performed on the collection day.

**Table 1:** Information on the particulate ^137^Cs monitoring campaigns^a^.

No.	Catchment Name	Longitude	Latitude	Area	^137^Cs inventory^b^	Particulate ^137^Cs monitoring period
		(°N)	(°E)	(km^2^)	(kBq m^−2^)	
1	Notegami	37.66	140.79	103	775	Sept.2014 – Jul.2017
2	Warabi	37.61	140.8	28	1490	Aug.2014 – Jul.2017
3	Haramachi	37.65	140.96	199	964	Dec.2012 – Dec.2018
4	Sakekawa	37.64	141.01	243	769	Aug.2014 – Jul.2017

^a^All the information can be found in our previous work.

^b^Inventory data were derived from Kato *et al*.

**Fig. 6:**
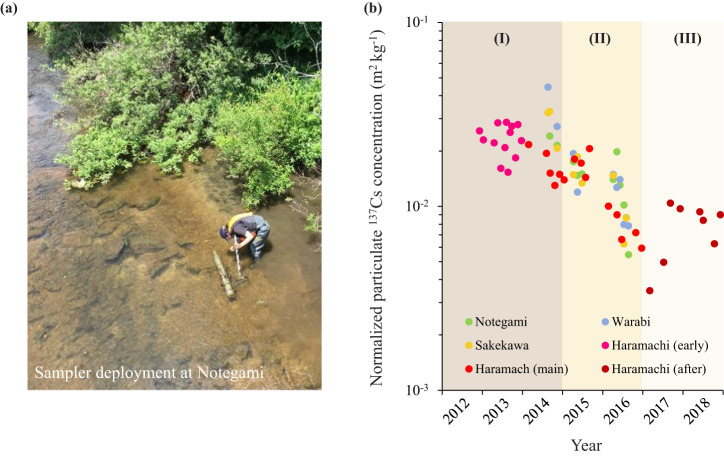
Particulate ^137^Cs monitoring in the decontaminated catchment. (**a**) Picture of field monitoring campaign at Notegami catchment. (**b**) Temporal variation of particulate ^137^Cs in rivers of the decontaminated catchments (Notegami, Warabi, Sakekawa, and Haramachi). Phase I, II, and III represent the early decontamination stage (2012–2014), main decontamination stage (2015–2016), and natural restoration stage (2017–2018), respectively. The measured data were normalized by the ^137^Cs inventory in the corresponding catchment (Table 1) to eliminate the effect caused by different inventories (i.e., ^137^Cs concentration dividing the corresponding ^137^Cs inventory). Date correction to the collection day and grain size correction were also performed.

Several studies^39,40^ have suggested that terrestrial ^137^Cs tend to tightly bind to fine sediments, which may need to consider the influence of grain size of the collected SS samples on the ^137^Cs measurement. A laser diffraction particle size analyzer (SALD-3100, Shimadzu Co., Ltd., Kyoto, Japan) was used to determine the grain size distribution of the dried SS samples. Referring to the analysis procedure described in previous work^27,41^, the specific surface area (*SSA*, m^2^ g^−1^) of the SS sample was measured, and then grain size correction was implemented for the measured ^137^Cs data. In addition, considering the difference in topsoil ^137^Cs inventories among four catchments, we also normalized the measured ^137^Cs data with the catchment inventories derived from the reconstruction map by Kato *et al*. to compare their decline trends. Fig. 6 illustrates the temporal variation in normalized particulate ^137^Cs concentrations at four monitoring sites of the Niida river basin.

## Data Records

The decontamination boundary map package consists of three maps, i.e., ordering decontamination maps for 2012, 2013, and 2014. Each map provides information on each specific decontamination region’s land use type and area. Table 2 lists the corresponding relationship between the numerical digit and land use type. All the maps are publicly accessible in the Shapefile format on the Environmental Radioactivity Research Network Center, University of Tsukuba website (https://www.ied.tsukuba.ac.jp/database/00155.html)^31^.

**Table 2:** Symbol ID and land use of the ordering decontamination maps in 2012, 2013 and 2014.

Year	Symbol ID	Land use type
2012	0	Agricultural land
	1	Residential land
	2	Grassland
	3	Waterside
	4	Facilities
	5	School
	6	Other
2013	0	Residential land
	1	Agricultural land
	2	Facilities
	3	Grassland
	4	Other
	5	Waterside
	6	School
2014	0	Agricultural land
	1	Grassland
	2	Waterside
	3	Residential land
	4	Facilities

The land use change package includes 667 satellite maps of the decontamination region with a spatial resolution of 10 m × 10 m and a temporal resolution of 16 days (few images are unavailable). Table 3 provides an overview of file contents. All satellite images in gridded GeoTIFF format are accessible on the Environmental Radioactivity Research Network Center, University of Tsukuba (https://www.ied.tsukuba.ac.jp/database/00148.html)^30^.

**Table 3:** Information of the file contents in the database about satellite images.

No.	Parameter Mnemonic	Column	Type	Priority	Note
1	DOI	1	Text	1	/
2	DID	2	Int	1	/
3	Ordering year	3	Int	1	/
4	LatDir	4	Text	1	/
5	Nsflag	5	Int	1	1 in NH, −1 in SH
6	xlat_min	6	Int	1	minimum latitude of decontamination regions
7	xlat_max	7	Int		maximum latitude of decontamination regions
8	LonDir	8	Text	1	/
9	Ewflag	9	Int	1	1 in EH, −1 in WH
10	xlong_min	10	Numeric	1	Minimum longitude of decontamination regions
11	xlong_max	11	Numeric	1	Maximum longitude of decontamination regions
12	Image name	12	Text	1	Median date of satellite image observation
13	Image yyyy	13	Int	2	/
14	Image mm	14	Int	2	/
15	Image dd	15	Int	2	/
16	Image xyear	16	Numeric	1	yyyy + (mm−1)/12 + (dd-1)/365
17	mean NDVI	17	Numeric	0	Image spatial resolution of 10 m

The concurrent river monitoring package involves river water monitoring and particulate ^137^Cs monitoring. The river monitoring datasets, including water discharge and SS concentration upstream (Notegami, 250872 records) during 2015–2018 and downstream (Haramachi, 631008 records) during 2013–2018, are publicly accessible on the website of the Environmental Radioactivity Research Network Center, University of Tsukuba (https://www.ied.tsukuba.ac.jp/database/00147.html)^29^. Table 4 presents the content of this database. The particulate ^137^Cs monitoring datasets, including samples from four monitoring sites (Notegami, Warabi, Haramachi, and Sekekawa), were shown on our website as well (https://www.ied.tsukuba.ac.jp/database/00146.html)^28^. Table 5 summarizes its content description.

**Table 4:** Information of the file contents in the database about river monitoring.

No.	Parameter Mnemonic	Column	Type	Unit	Priority	Note
1	DOI	1		Text	1	
2	DID	2		Int	1	
3	station	3		Text	0	
4	yyyy	4		Int	2	
5	mm	5		Int	2	
6	dd	6		Int	2	
7	hh	7		Int	2	
8	min	8		Int	2	
9	xyear	9		Numeric	1	yyyy + (mm−1)/12 + (dd−1)/365
10	LatDir	10		Text	1	
11	Nsflag	11		Int	1	1 in NH, −1 in SH
12	xlat	12		Numeric	1	
13	LonDir	13		Text	1	
14	Ewflag	14		Int	1	1 in EH, −1 in WH
15	xlong	15		Numeric	1	
16	altdepflag	16		Int	1	1 above ground, −1 below ground
17	sampdep	17	m	Numeric	1	
18	sample type	18		Text	1	
19	Water discharge	19	m^3^ s^−1^	Numeric	0	
20	Suspended sediment concentration	20	g L^−1^	Numeric	0	
21	Uncertainty of suspended sediment concentration	21	g L^−1^	Numeric	0

**Table 5:** Information of the file contents in the database about particulate ^137^Cs monitoring.

No.	Parameter Mnemonic	Column	Type	Unit	Priority	Note
1	DOI	1		Text	1	
2	DID	2		Int	1	
3	station	3		Text	0	
4	decontmianted?	4		Text	0	yes or no
5	yyyy	5		Int	2	
6	mm	6		Int	2	
7	dd	7		Int	2	
8	xyear	8		Numeric	1	yyyy + (mm−1)/12 + (dd−1)/365
9	LatDir	9		Text	1	
10	Nsflag	10		Int	1	1 in NH, −1 in SH
11	xlat	11		Numeric	1	
12	LonDir	12		Text	1	
13	Ewflag	13		Int	1	1 in EH, −1 in WH
14	xlong	14		Numeric	1	
15	altdepflag	15		Int	1	1 above ground, −1 below ground
16	sample type	16		Text	1	
17	Specific Surface Area (SSA)	17	m^2^ g^−1^	Numeric	0	
18	Nuclide	18		Text	0	^137^Cs
19	Activity	19	Bq kg^−1^	Numeric	0	
20	Uncertainty	20	Bq kg^−1^	Numeric	0	

## Technical Validation

**Fig. 7:**
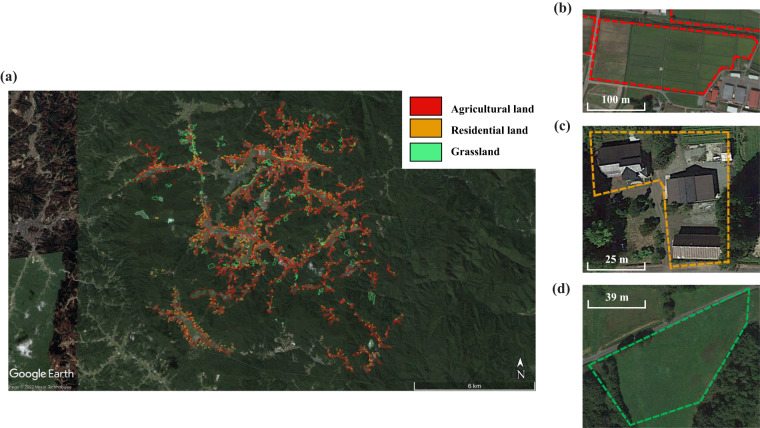
Validation of outlined decontaminated regions in the Niida river basin. (**a**) full picture of decontaminated regions in the Niida river basin with major land use types. The examples of validation of three major land use types, including (**b**) agricultural land, (**c**) residential land, and (**d**) grassland in the decontaminated regions by using images in Google Earth.

Strict quality control was used in the vector decontamination outline to ensure consistency with the original paper map. In addition, a comparison between the outlined decontaminated regions and satellite images shown on Google Earth was also implemented to verify the correctness of their land use types (procedure shown in Fig. 3). In Fig. 7, we provide an example to show how we validated the land-use in vector maps by overlapping the images in Google Earth. This process was repeated by three co-authors and was confirmed for map quality assurance.

**Fig. 8:**
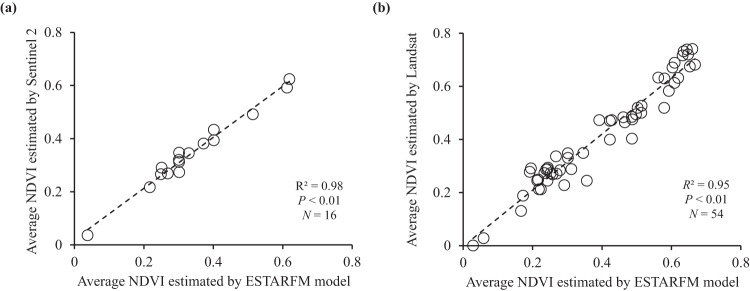
Regression analysis of average NDVI estimated by the ESTARFM-based images with average NDVI estimated by (**a**) Sentinel 2 images and (**b**) Landsat images during the same period.

To check the reliability of the NDVI calculated using the ESTARFM-based images, we compared the average NDVI in decontaminated regions obtained from Sentinel 2 images and ESTARFM images acquired during the same period. Linear regression analysis showed a good agreement with a high *R*^2^ of 0.98 (*P* < 0.01, *n* = 16) between the two groups of datasets (Fig. 8a). In addition, we downloaded the available Landsat satellite images (Landsat 5/7/9, spatial resolution of 30 m) from the USGS (https://earthexplorer.usgs.gov/) and estimated the average NDVI value in the decontaminated regions during 2011–2018. Similarly, we performed a linear regression analysis of the average NDVI obtained from Landsat and ESTARFM images during the same period. R^2^ also showed a good correlation between the two datasets with value of 0.95 (*P* < 0.01, *n* = 54, Fig. 8b). Table 6 summarizes the basic information (resolution, period, and fitting parameters) of NDVI calculated from different satellite images. These results demonstrate the reliability and feasibility of the estimated NDVI mean value using ESTARFM-images.

**Table 6:** Basic information of NDVI comparison among different satellite images.

No.	ESTARFM	Sentinel 2	Landsat
Spatial resolution	10 m	10 m	30 m
Available period	2011–2018	2016–2018	2011–2018
Available images	165	16	54
Fitting parameter (a)^1^	/	0.96	1.06
Fitting parameter (b)^1^	/	0.02	0
R^2^	/	0.98	0.95

1. *NDVI*_Sentinel 2/Landsat = _a* *NDVI*_ESTARFM_ + b.

The uncertainties of all the river monitoring datasets were carefully considered in this study. Specifically, we used the following strategy for the uncertainty assessment:

1. For the correctly recorded water level and turbidity datasets, we did not consider the uncertainty of the value because the 95% confidence intervals of the conversion curves (i.e., water level-discharge curve and turbidity-SS concentration curve) are typically narrow.
2. For the missing water level data, it is difficult to assess the uncertainty of those filling data because of the unavailability of error information in the water level datasets provided by the Fukushima prefecture’s official monitoring network. However, these official water level monitoring data should remain reliable because river monitoring campaigns organized by the official river monitoring network were implemented under quality control.
3. For the missing turbidity data, the 95% confidence interval of the discharge-SS concentration curve in the given year (power equation) was used as the uncertainty of the filling data.
4. For the particulate ^137^Cs dataset, the main uncertainty was attributed to the measurement. Therefore, the statistical fluctuations in radiation measurement were thought to be uncertain.

In the river monitoring datasets (Fig. 5c), the slightly higher SS concentration in baseflow in 2016 can be attributed to the high completion of governmental decontamination work (about 50%) in 2016^32^. Integrated with the lower NDVI values and the rapid particulate ^137^Cs concentration decline in 2016, we suggest that the stronger land perturbation in the decontaminated regions led to more decontaminated sediment transfer from the upstream regions to the river system, which in turn enhanced the availability of river suspended sediment.

## Data Availability

Not applicable in this work.
